# Supplementary material for: Unraveling the Folding Dynamics of DNA Origami Structures
Source: Small. 2025 Oct 19;21(49):e04855. doi: 10.1002/smll.202504855 (PMC12696799; doi:10.1002/smll.202504855)
Supplement: Supplementary file 1 — Supporting Information [file SMLL-21-e04855-s001.pdf]

NANO · MICRO
small

Supporting Information

for *Small*, DOI 10.1002/smll.202504855

Unraveling the Folding Dynamics of DNA Origami Structures

*Meysam Mohammadi-Zerankeshi, James Houston, Ogochukwu K.U. Elisha-Wigwe, Abi Sachi and Alexander E. Marras**

Supplemental Information

Unraveling the Folding Dynamics of DNA Origami Structures

*Meysam Mohammadi Zerankeshi, James Houston, Ogochukwu K.U. Elisha-Wigwe, Abi Sachi, Alexander E. Marras**

Walker Department of Mechanical Engineering, University of Texas at Austin, Austin 78712, Texas, United States

*Corresponding author

Table of Contents

Supplemental Methods ____ 2

Controlled reactions ____ 2

Real-time fluorometry experiments ____ 2

Theoretical energy calculations ____ 3

Supplemental Results: Folding Experiments and Energy Calculations ____ 3

Cadnano designs ____ 13

Supplemental References ____ 22

Supplemental Methods

Controlled reactions

The temperature-dependent assembly of 24 hb-rigid and flex DNA origami structures was conducted based on their real-time fluorometry curves. For these experiments, 50 µL of reaction mixture was aliquoted into PCR tubes, heated to 85 °C, and then cooled following the same thermal ramp protocol used in Figure 2 (85 to 25 °C, -0.2 °C per step, with a 13-second hold at each temperature). Once the samples reached the target temperatures, they were removed from the thermal cycler and quenched in liquid nitrogen. For the constant-temperature annealing protocol, samples were heated to the desired temperature and maintained for a specified duration before being similarly quenched in liquid nitrogen. After preparation, all samples were thawed and promptly analyzed using gel electrophoresis or TEM.

Real-time fluorometry experiments

To monitor fluorescence intensity changes, SYBR Green was added to the folding tubes of various DNA origami structures at a ratio of one molecule per 900 DNA base pairs. Each DNA origami sample consisted of 12 µL of the same reaction components as the folding assembly, with the addition of SYBR Green, and was aliquoted into PCR tubes. Additionally, reference reactions were prepared, including (i) only the scaffold (excluding staples), (ii) only staples (excluding the scaffold), (iii) only buffer (excluding both scaffold and staples), and (iv) a DNA hairpin with a melting temperature of 74°C of the following sequence:

5′-TCAACATCAGTCTGATAAGCTACACGAGATCAGACTGATGTTGA-3′

DNA hairpin acts as a reference control to mimic a solution with a temperature-independent content of DNA base pairs. The rationale for including this reference follows the approach of Sobczak *et al.*,[1] where monitoring the hairpin reaction helped to separate intrinsic effects on SYBR Green fluorescence, such as the temperature-dependent affinity of SYBR Green for DNA, from fluorescence changes that reflect actual base pair formation in the sample. This allowed us to isolate the component of the fluorescence signal specifically arising from DNA origami folding, as opposed to nonspecific dye behavior. The signal from scaffold only suggests secondary structure formation in the temperature range of 40-60 °C, with little to no change observed below 40 °C, indicating that no more base pairs were formed at lower temperatures. Unlike the other references, the staple-only sample showed a more pronounced change in fluorescence as the temperature decreased, suggesting that staple strands engaged in progressive base pairing throughout the full temperature range, and hence, was subtracted from the origami folding reaction for rate of folding calculation, similar to observed behavior in Ref. [1]

Different thermal ramping protocols were tested to optimize the cooling rate for maximizing the yield of all studied DNA origami structures while covering a broad temperature range to capture their assembly behavior. Temperature was decreased from 85 to 25 °C at rates of -0.1 °C per step with hold times of 17, 72, and 312 seconds, as well as -0.2 °C per step with a 13-second hold. Figure S1 presents the real-time fluorometry results for all tested ramps. To further assess the impact of ramping rates, samples were analyzed using 2% agarose gel electrophoresis after each thermal protocol (Figure S3). For such thermal ramp to cover the range of 85-25 °C, the results indicate that slower cooling rates led to minimal structure formation across all designs, and increasing the cooling rate improved the yield. Notably, the -0.2 °C per step with a 13-second hold produced the highest yield for nearly all structures. For the focused thermal ramps, structures were heated only to the temperature at which fluorescence intensity began to increase, then gradually cooled at a rate of -0.1 °C per step, with a 13-second hold at each temperature, until the signal stabilized. For example, one such ramp spanned from 68 °C to 41 °C for 30 hb.

Unfolding investigations were conducted after the cooling ramp for all tested thermal protocols, using a heating rate identical to the cooling rate (Figure S2). The real-time fluorometry unfolding plots of samples that did not successfully form the desired DNA origami structures exhibited a unique feature: they were nearly identical and showed fluorescence intensity changes only at very high temperatures (~80 °C), suggesting aggregation dissociation. In contrast, the optimized thermal ramp, which led to well-formed DNA origami structures, displayed distinct unfolding behaviors for different designs. These findings indicate that real-time fluorometry can be used to assess whether a given thermal ramp successfully facilitates proper DNA origami folding or induces undesired interactions, such as scaffold-scaffold, staple-staple, or staple-scaffold associations.

Theoretical energy calculations

The theoretical loop, hybridization and binding energies were calculated using the code in Ref. [2] In this regard, the caDNAno files of structures were used in 'Read mode'. Binding energy was calculated from a fixed 100 nM staple concentration and 50 °C temperature and based on the Eq. S1. [2,3] At this concentration and a modeling temperature of 50 °C, ΔG_{bind} was calculated as +10.3 kcal/mol using the expression in Eq. 1. In this equation, R is the gas constant = 1.987 (cal/mol.K), T is temperature in kelvin and C is the molar concentration of the staple. Because staple concentration was held constant, ΔG_{bind} does not contribute to relative differences in folding behavior between designs or staple lengths and was assumed the same for all staples.

$$\Delta G = -RT \ln C \qquad \text{Eq. S1}$$

In this Equation, R is the gas constant (1.987 cal/(mol.K)), T is the temperature (K) and C is the molar concentration of the staple strand.

Supplemental Results: Folding Experiments and Energy Calculations

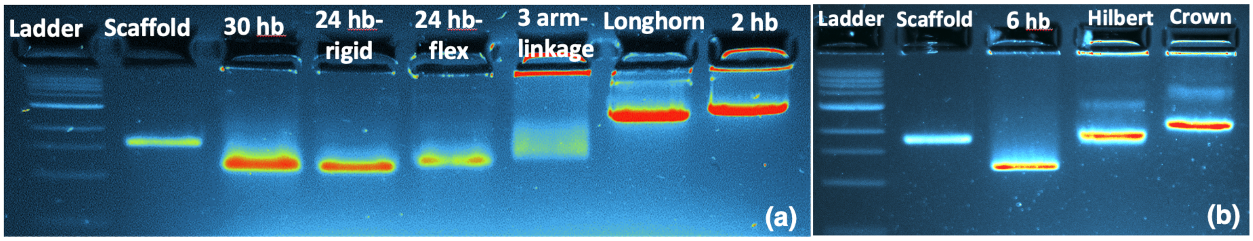

Figure S1. Agarose gel electrophoresis of the studied structure including (a) 30 hb, 24 hb-rigid, 24 hb-flexible, 3-arm linkage, Longhorn and 2 hb and (b) 6 hb, Hilbert, and Crown, after the same thermal ramp used in the fluorometric assay experiment,

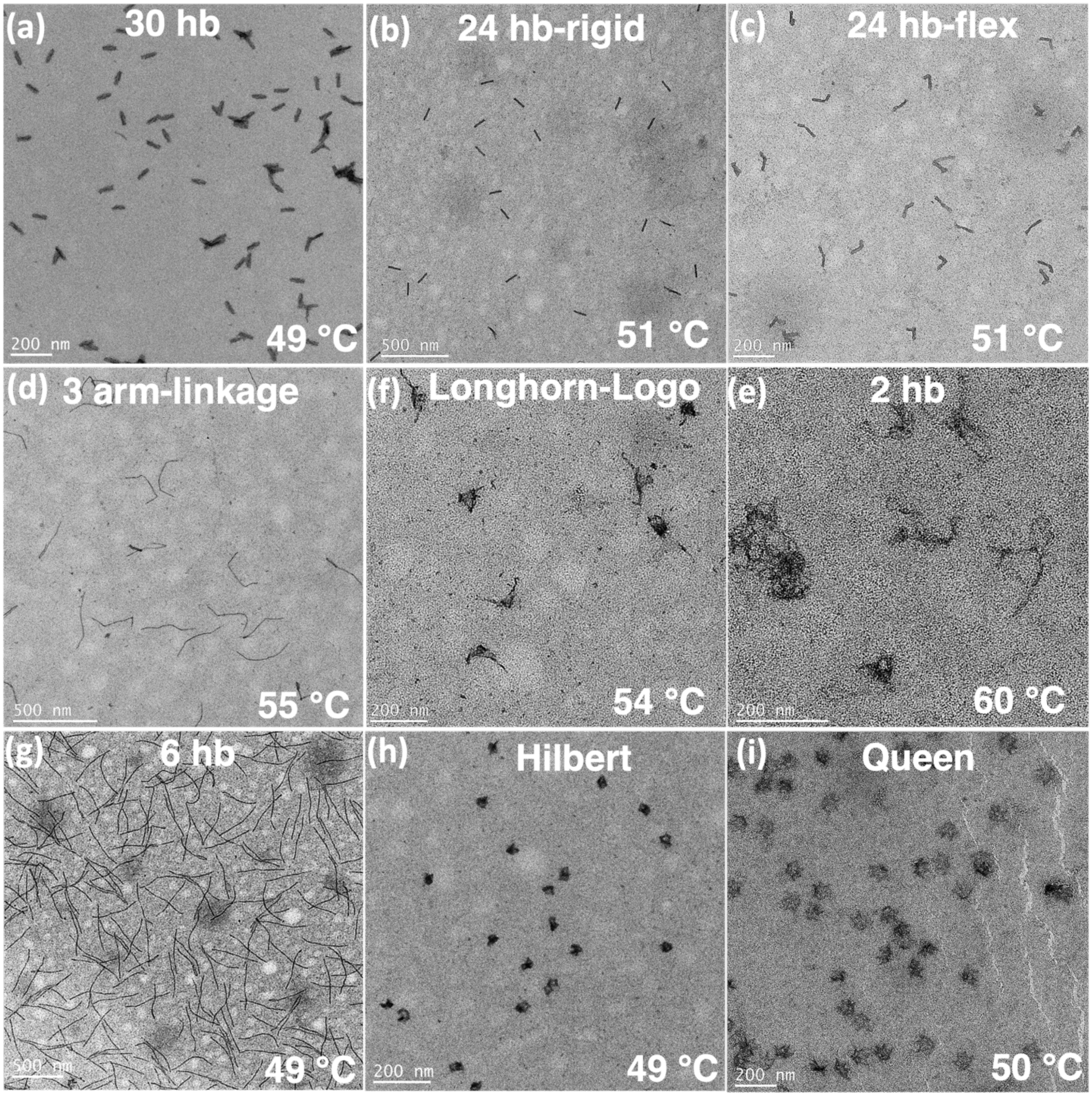

Figure S2. TEM images of the studied structures before their T_{unfold}. Structures were folded first with the same thermal ramp in Figure 2 and then heated to the associated temperatures with the same ramp and were shock-frozen once they reached these temperatures.

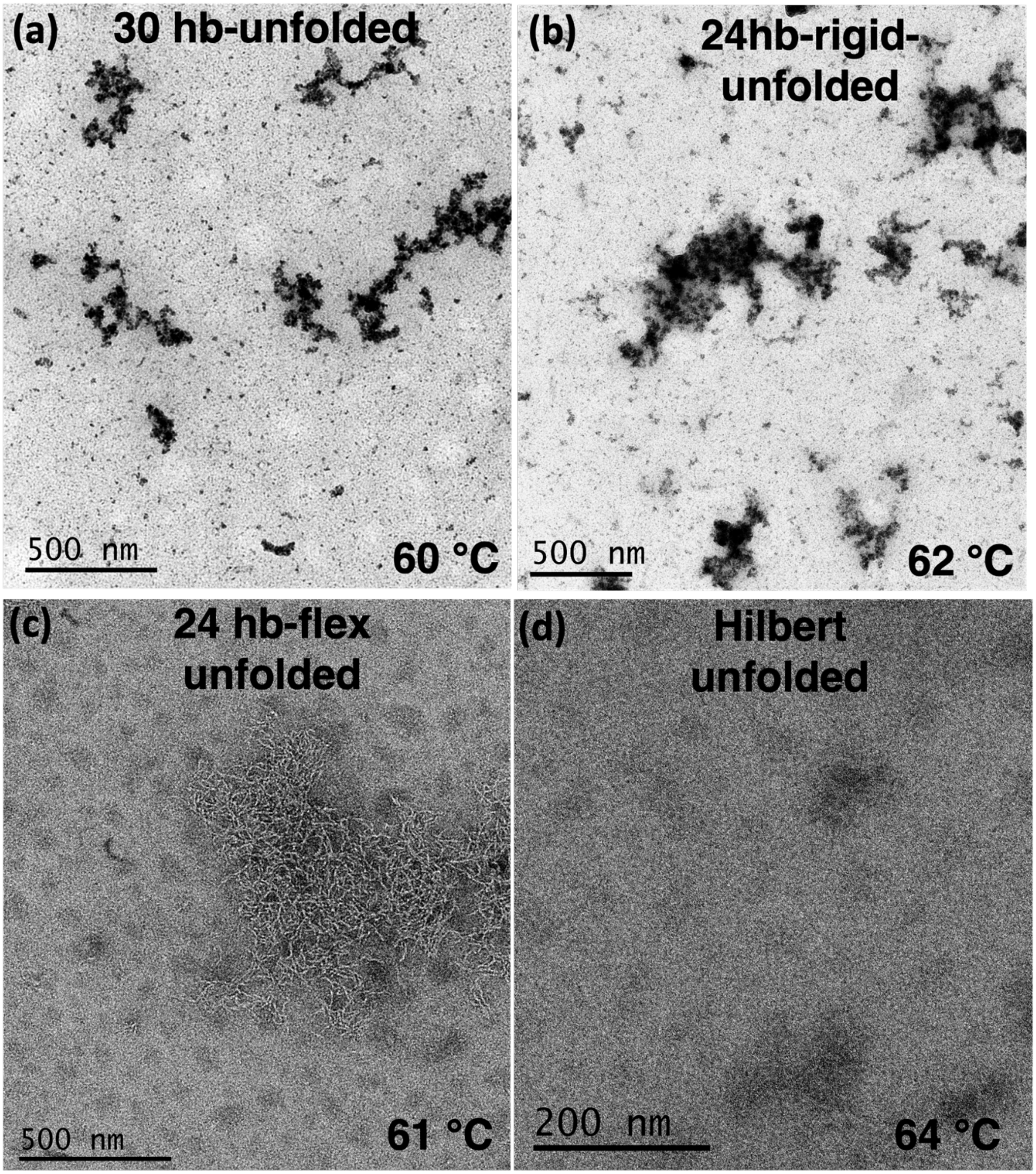

Figure S3. TEM images of the studied structures at temperatures above their T_{unfold}. Structures were folded first with the same thermal ramp in Figure 2 and then heated with the same ramp and were shock-frozen once they reached these temperatures.

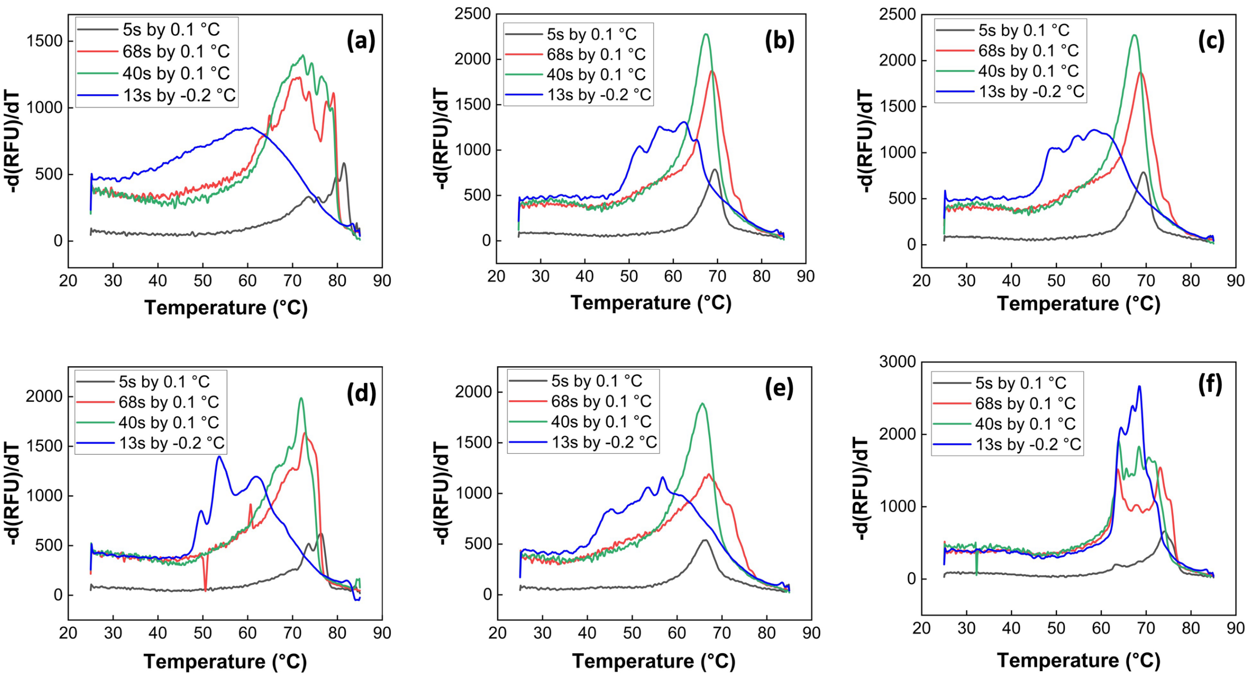

Figure S4. Real-time fluorometry results for (a) 30 hb, (b) 24 hb-rigid, (c) 24 hb-flex, (d) 3-arm linkage, (e) Longhorn, and (f) 2 hb, after annealing from 85 to 25 °C with different rates such as decreasing the temperature by 0.1 °C and staying at each temperature for 312 seconds (blue), 72 seconds (red), and 17 seconds (black) as well as dropping the temperature by –0.2 °C with 12 seconds time for each temperature during cooling (green). These results are before staple reduction from the DNA origami object data.

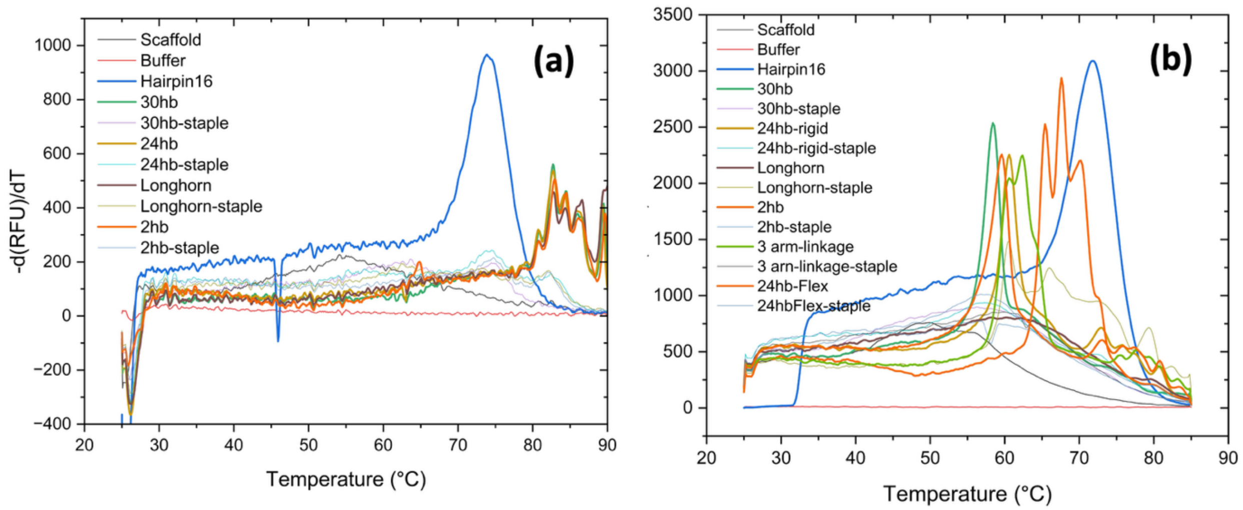

Figure S5. Unfolding real-time fluorometry result of different structures that were fold previously by cooling from 85 to 25 °C by (a) -0.1 °C for 312 seconds at each temperature (longest thermal ramp) and (b) -0.2 °C for 13 seconds, before staple reduction from DNA origami object.

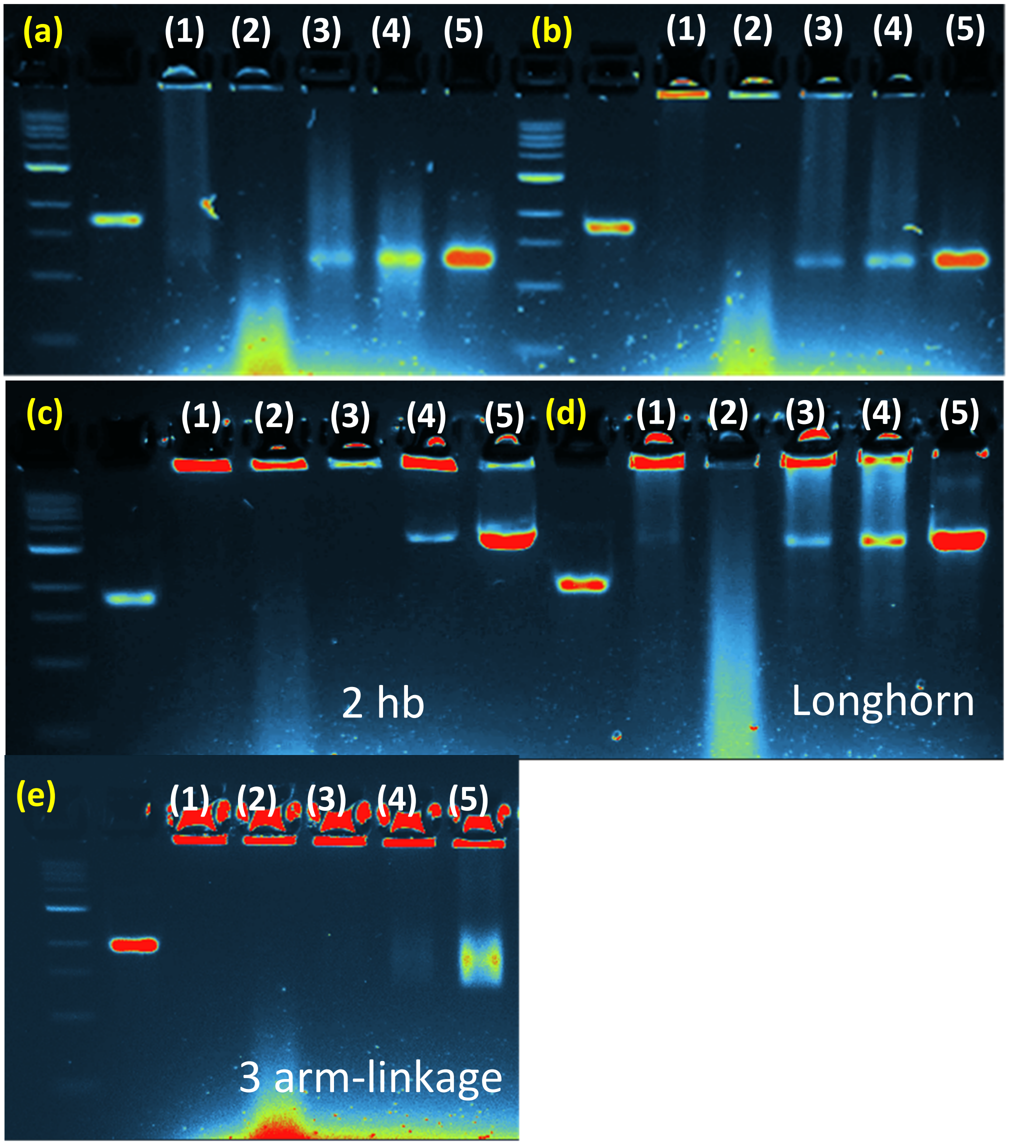

Figure S6. 2% Agarose gel electrophoresis of (a) 30 hb, (b) 24 hb-rigid, (c) 2 hb, (d) Longhorn and (e) 3-arm linkage structures after cooling from 85 to 25 °C with decreasing the temperature by -0.1 °C and heating at each temperatures for (1) 312 seconds, (2) 72 seconds, (3) 17 seconds, and (4) temperature drop of -0.2 °C for 12 seconds. The first lane in all gel images is 1kbp ladder and the second lane is scaffold as the control.

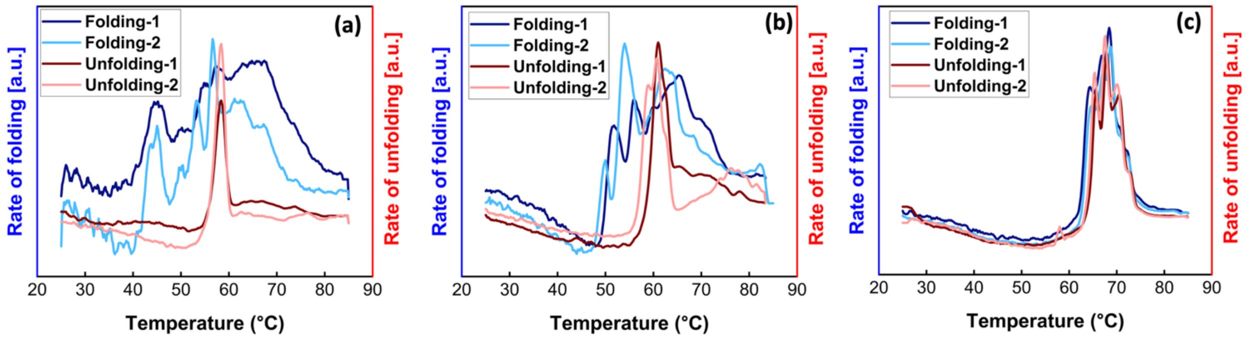

Figure S7. Replicates of folding and unfolding real-time fluorometry assay of (a) 30 hb, (b) 3 arm-linkage and (c) 2 hb.

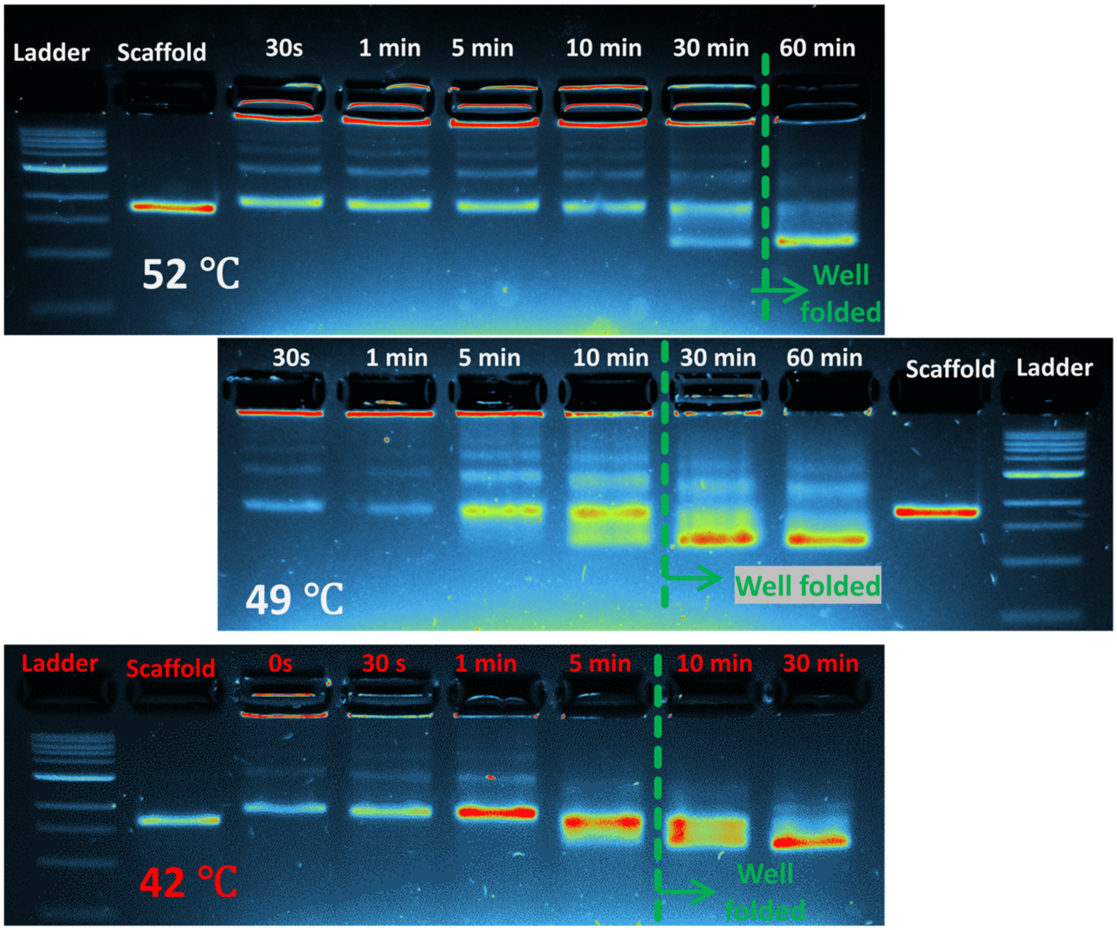

Figure S8. Constant temperature annealing of 30 hb DNA origami structure at 52, 49 and 42 °C for different time points. 30 hb was ultimately folded in all temperatures, indicating the negligible role of hybridization energy at those temperatures. Decreasing the temperature fastened the folding, where a decent yield was achieved at 42 °C, after 10 min.

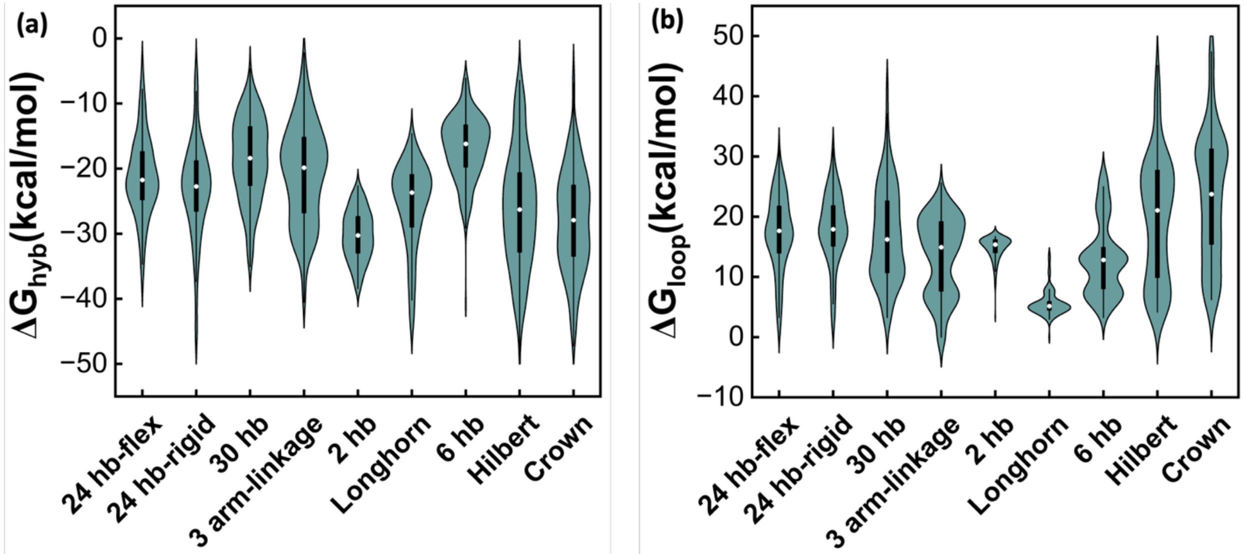

Figure S9. (a) Hybridization and (b) loop formation energies for all staples in all studied DNA origami structures obtained by using the computation method introduced in Ref[2]. The dark rectangles represent the 25-75% percentile of the data, the bars show the 1.5 interquartile range, and the circle is the median.

Table S1. The staple length and number of crossovers per staple relationship with free energy changes during folding of different clusters of 3 arm-linkage structure that was divided into three separate arms, obtained by the platform in Ref [2]. Arms 1, 2 and 3 are shown in the caDNAno design in Figure S13d.

**Cluster**	**Average staple length**	**Average number of crossovers per staple**	**Average ΔG_{hyb}** (kcal/mol)	**Average ΔG_{loop}** (kcal/mol)	**Average ΔG_{total}** (kcal/mol)
Arm 1-original	31.65	2.21	-20.42	11.65	1.61
Arm 1-longer	37.21	2.74	-24.43	13.70	-0.35
Arm 2-original	31.65	2.28	-21.06	14.31	3.63
Arm 2-longer	37.22	2.89	-24.95	16.83	2.25
Arm 3-original	32.03	2.26	-21.27	12.57	1.68
Arm 3-longer	37.91	2.77	-24.74	14.88	0.52

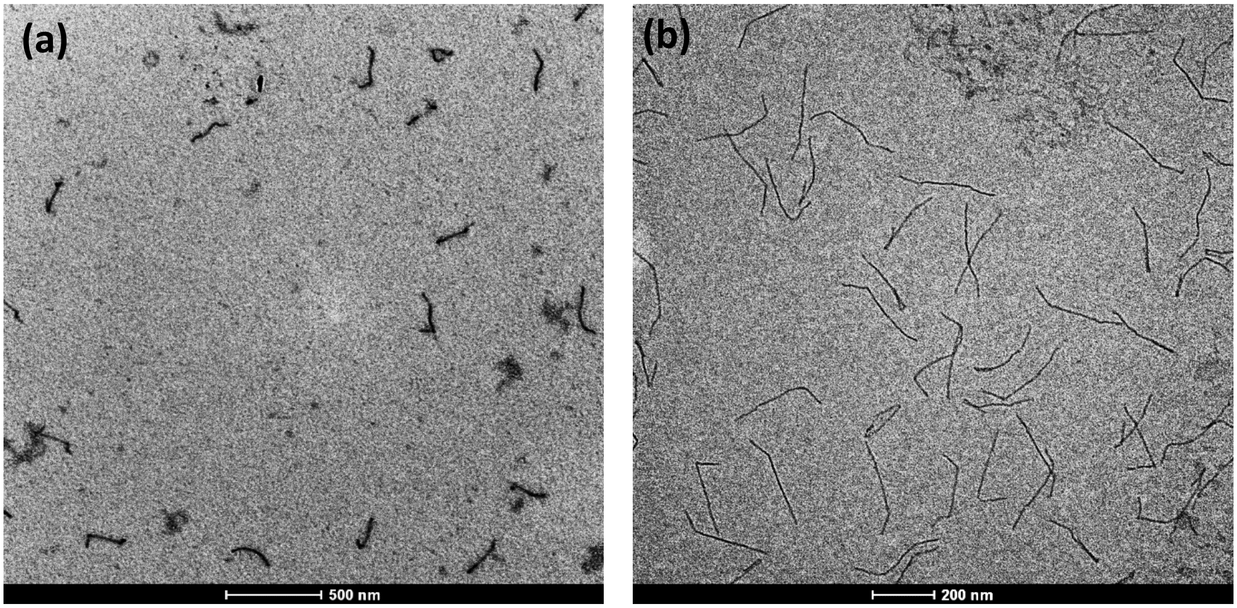

Figure S10. TEM images of 3 arm-linkage: (a) the design with longer sections of staples and (b) original design.

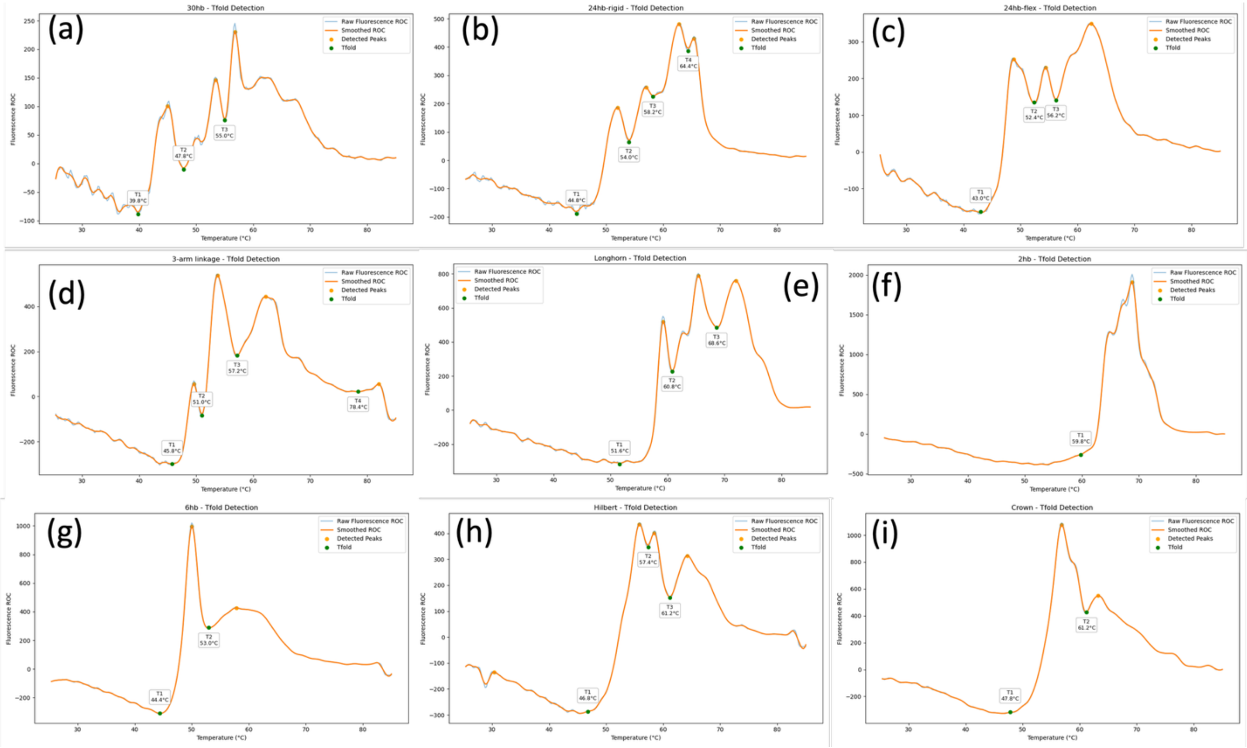

Figure S11. Python code output of graphs for all tested structures that calculate the T_{fold} from the fluorescence data and by comparing it to the theoretical T_{fold} of structure staples, can calculate the role of cooperativity.

This code also offers a manual override of T_{fold} in the case that noisy data leads to an inaccurate automatic T_{fold} selection. The code will output the percent of cooperativity for each design. The source code for the cooperativity calculations is available under an open-source license at: https://github.com/NanoassemblyLabUT/cooperativity-analyzer

Table S2. ΔG_{hyb} and ΔG_{loop} mean values for each structure obtained from Ref [2].

Structure	ΔG_{hyb} mean (kcal/mol)	ΔG_{loop} mean (kcal/mol)
**Longhorn**	-25.5	5.1
**3-arm linkage**	-20.6	13.4
**24 hb-flex**	-21.2	17.4
**30 hb**	-18.5	17.0
**24 hb-rigid**	-23.1	18.0
**2 hb**	-30.2	14.7
**6 hb**	-16.7	12.8
**Hilbert**	-26.1	19.8
**Crown**	-27.9	23.6

Table S3. Folding yield and migration distance of the three studied designs in Figure 7

Structure	Sample	Band migration (px)	Intensity (%)
**24 hb-flex**	0	143.47	25.51
	30 s	148.47	58.05
	1 min	149.47	68.49
	5 min	162.47	78.94
	10 min	180.47	80.6
	30 min	180.47	83.31
**24 hb-rigid**	0	130.85	63.52
	30 s	174.85	69.88
	1 min	181.85	84.24
	5 min	179.85	87.59
	10 min	182.85	96.5
	30 min	187.85	97.86
**30 hb**	0	124.53	63.52
	30 s	168.53	69.88
	1 min	175.53	84.24
	5 min	173.53	87.59
	10 min	176.53	96.5
	30 min	181.53	97.86

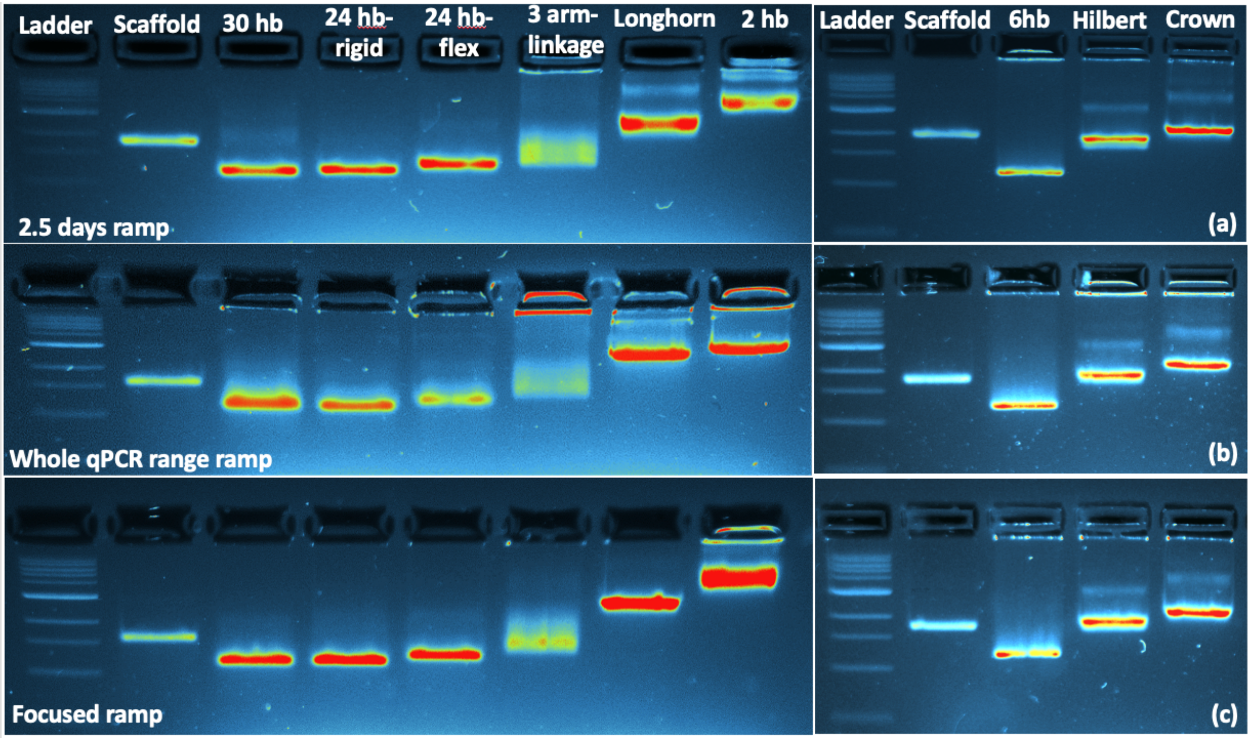

Figure S12. Agarose gel electrophoresis results in different structures after conducting a) 2.5 days thermal ramp, b) the real-time fluorometry ramp used in Figure 2 and c) focused thermal annealing at real-time fluorometry peaks regions and shock-freezing them after the last peak. The yield of the structures based on these gel images are presented in Figure 8.

Cadnano designs

(a)

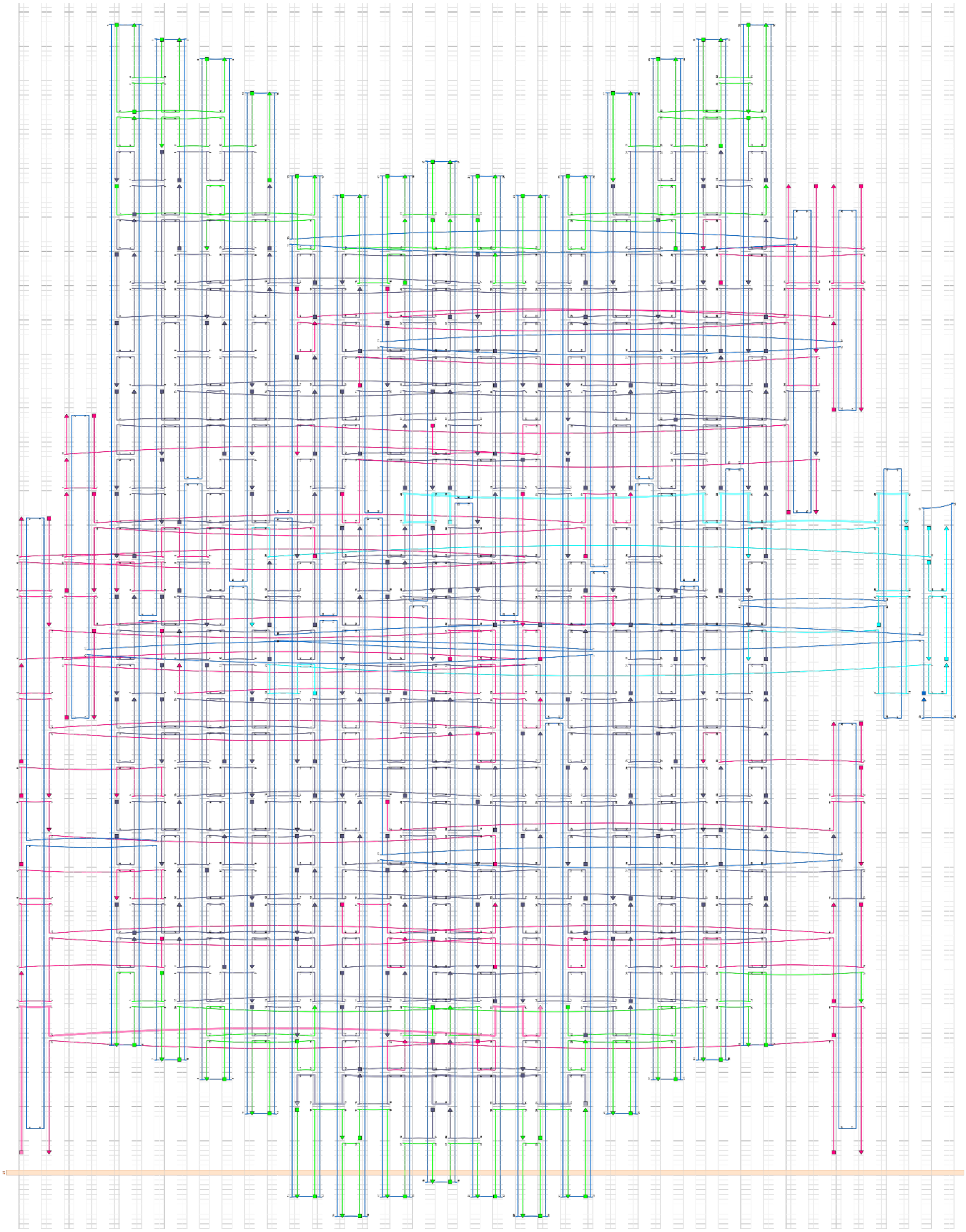

(b)

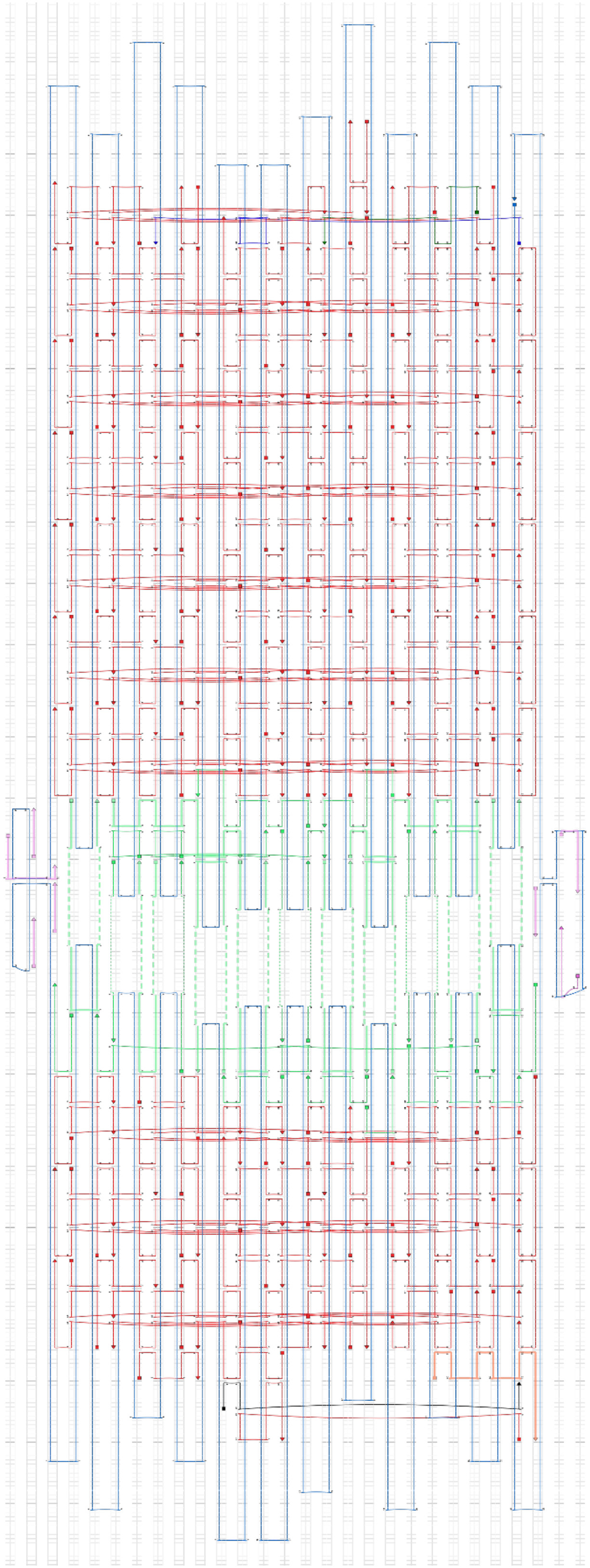

(c)

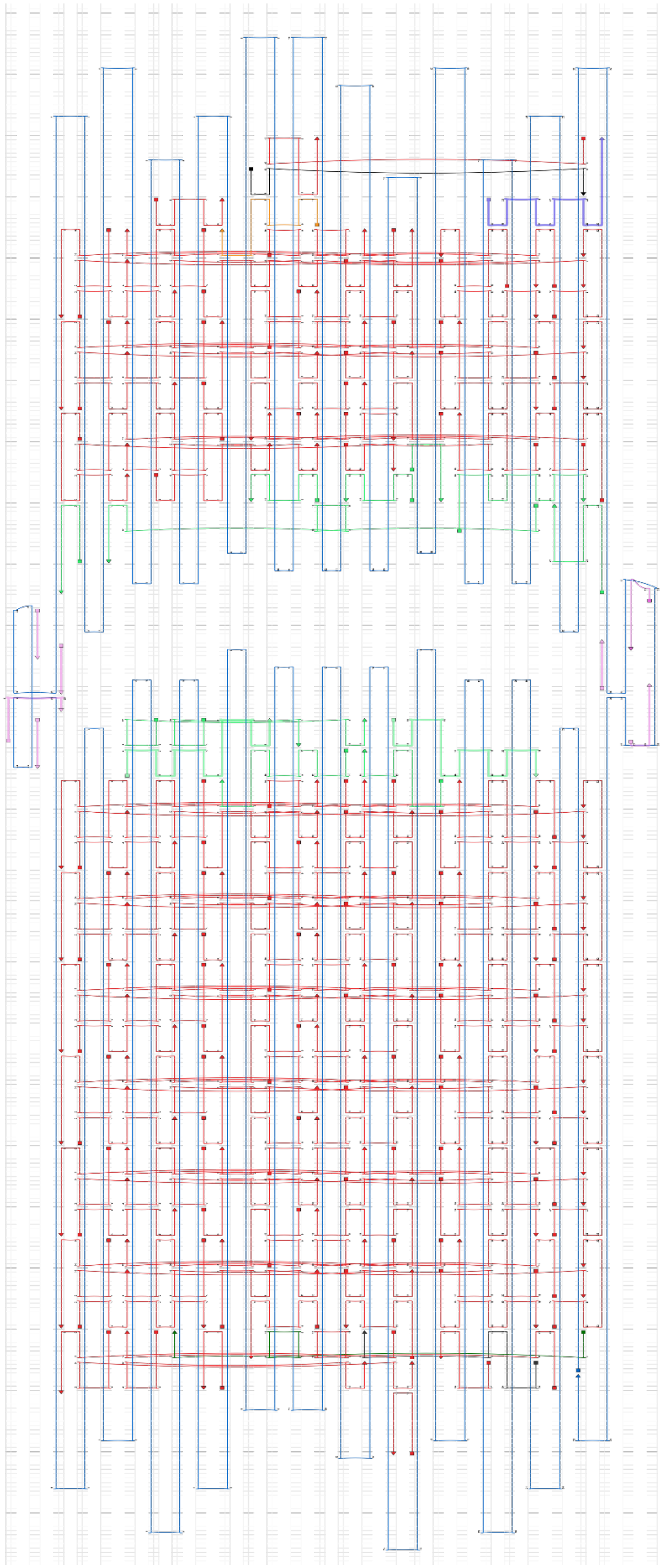

(d)

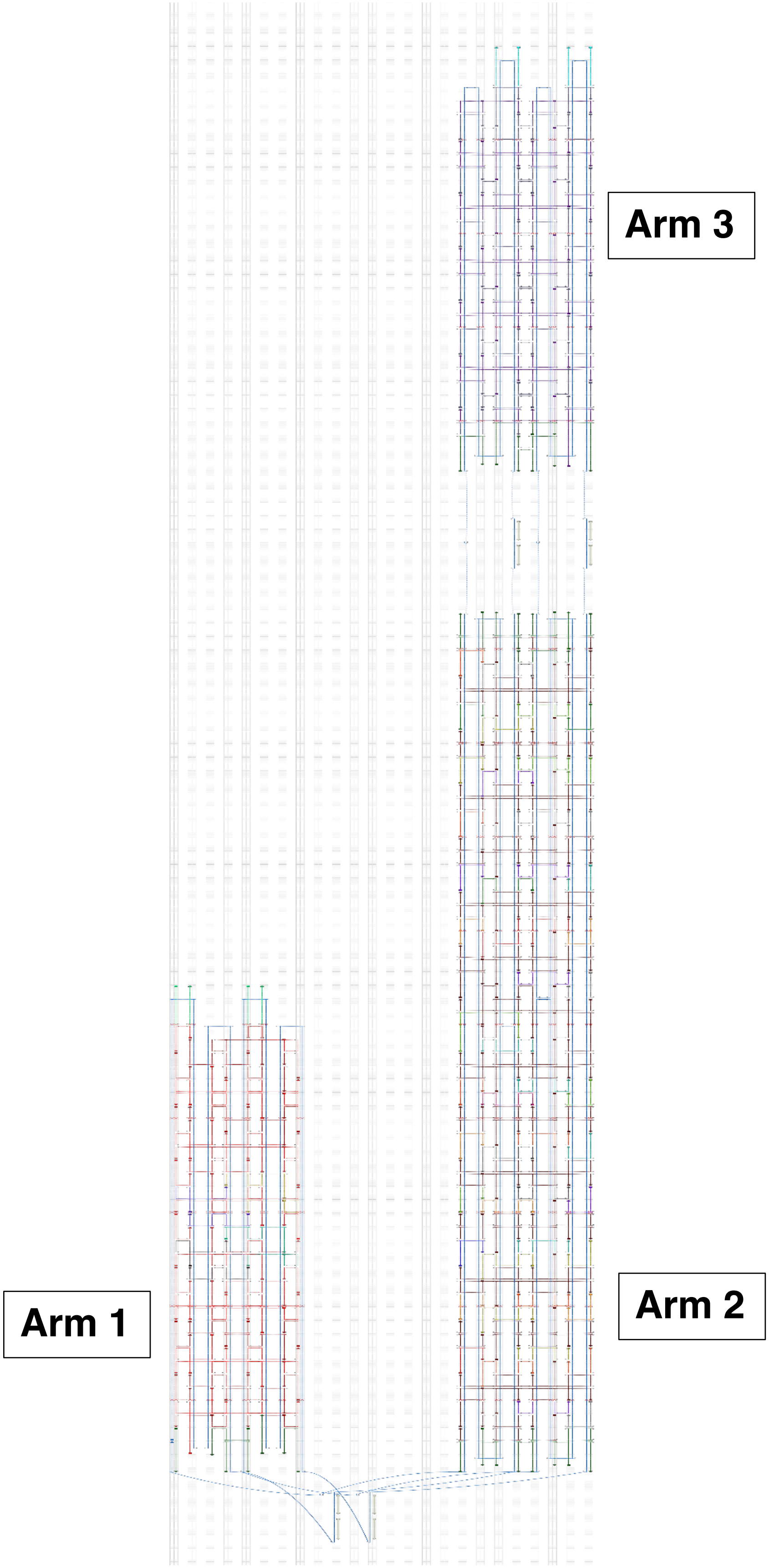

(e)

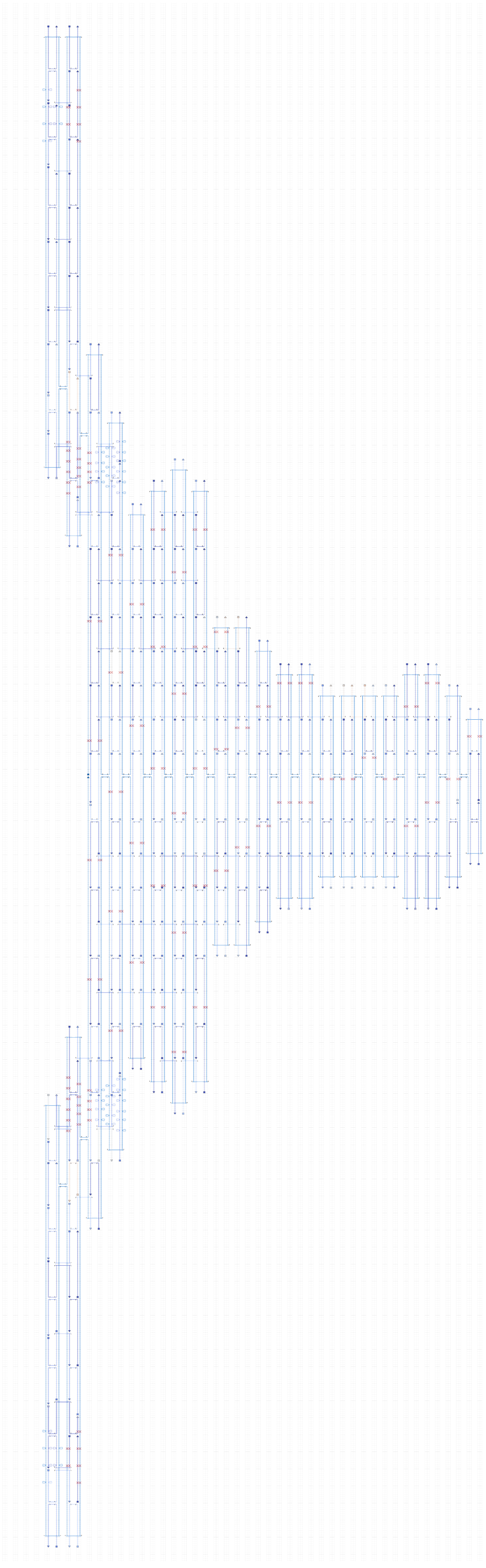

(f)

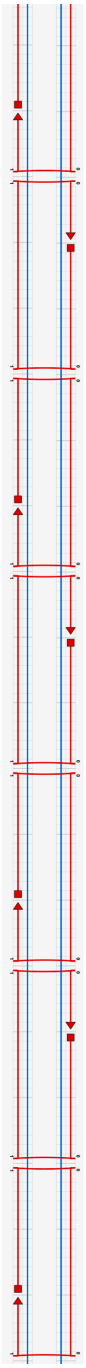

(g)

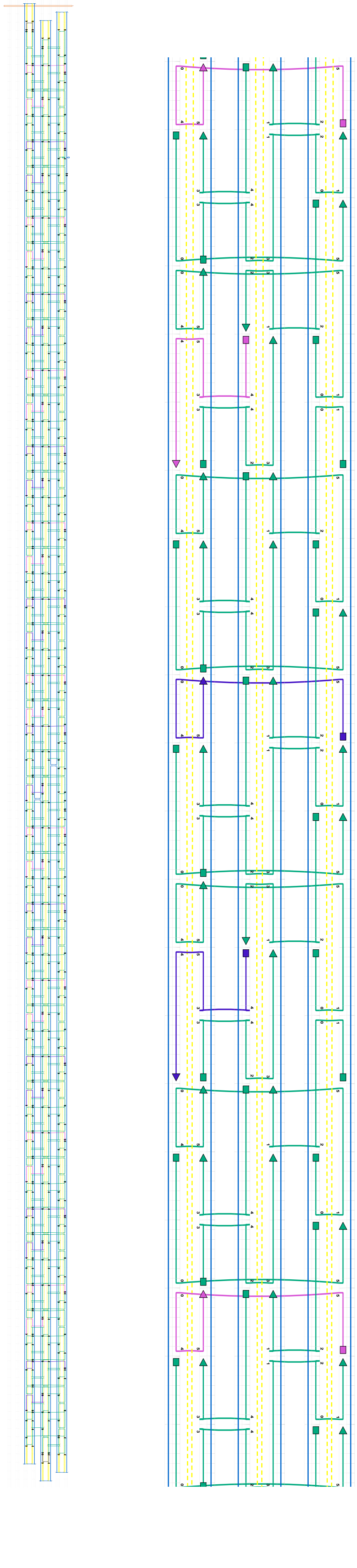

(h)

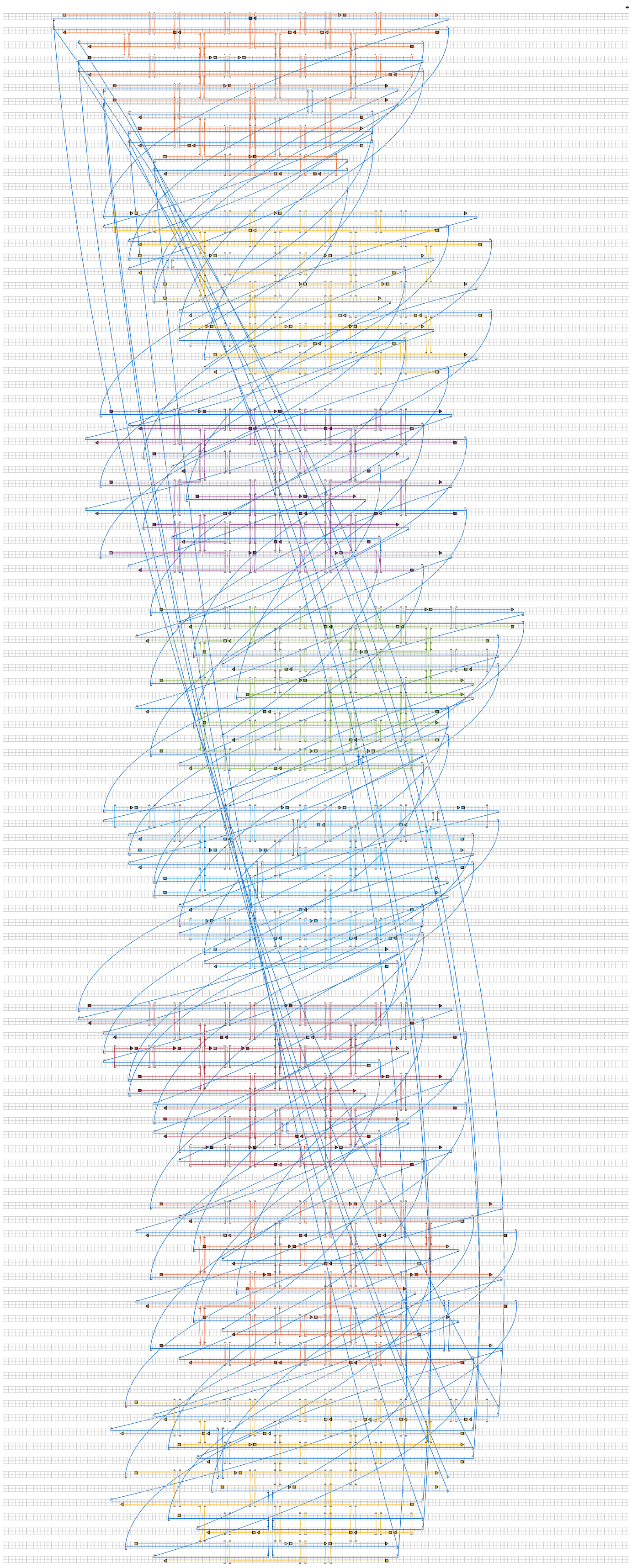

(i)

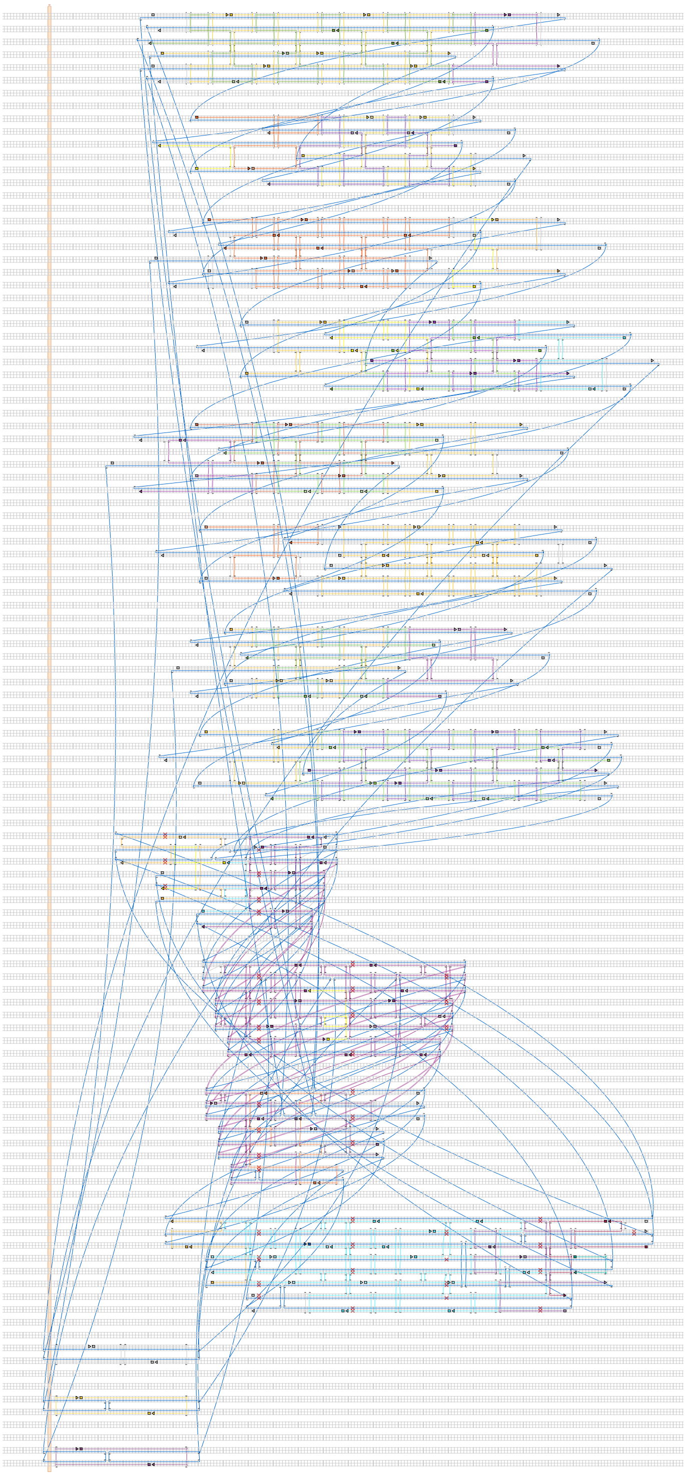

Figure S13. caDNAno designs for the studied DNA origami objects: (a) 30 hb, (b) 24 hb-rigid, (c) 24 hb-flex, (d) 3 arm-linkage, (e) Longhorn, and (f) cross-section of 2 hb, (g) 6 hb, (h) Hilbert and (i) Crown.

Supplemental References

1] S. M. Douglas, A. H. Marblestone, S. Teerapittayanon, A. Vazquez, G. M. Church, W. M. Shih, *Nucleic Acids Res.* **2009**, *37*, 5001.

[2] T. Aksel, E. J. Navarro, N. Fong, S. M. Douglas, *Proc. Natl. Acad. Sci.* **2024**, *121*, 2406769121.

[3] K. E. Dunn, F. Dannenberg, T. E. Ouldridge, M. Kwiatkowska, A. J. Turberfield, J. Bath, *Nature* **2015**, *525*, 82.
