# Supplementary material for: Unraveling the Folding Dynamics of DNA Origami Structures
Source: Small. 2025 Oct 19;21(49):e04855. doi: 10.1002/smll.202504855 (PMC12696799; doi:10.1002/smll.202504855)
Supplement: Supplementary file 2 — Supporting Information [file SMLL-21-e04855-s002.zip › Raw data-R3.pdf]

Raw Information

Unraveling the Folding Dynamics of DNA Origami Structures

*Meysam Mohammadi Zerankeshi, James Houston, Ogochukwu K.U. Elisha-Wigwe, Abi Sachi, Alexander E. Marras**

Walker Department of Mechanical Engineering, University of Texas at Austin, Austin 78712, Texas, United States

*Corresponding author email: amarras@utexas.edu

1. **Real-time fluorometry data.** The derivative of these raw intensity data are shown in Figure 2 to identify folding peaks. The raw real-time fluorometry from qPCR Excel sheet file for all data is also uploaded, so the melting and folding curves can be plotted.

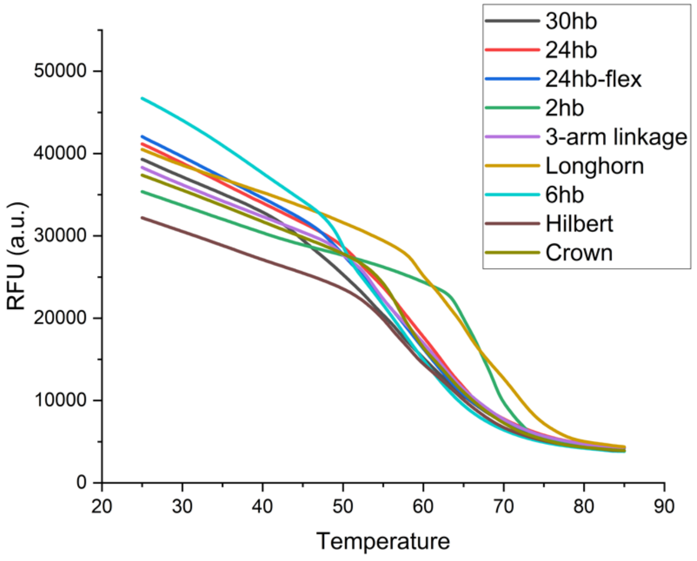

Raw data- DNA origami folding qPCR raw curves

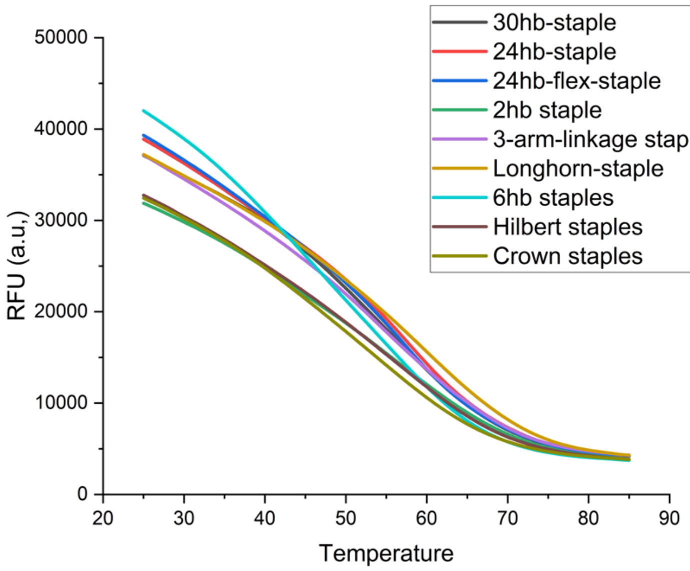

Raw data- DNA origami staples only folding qPCR raw curves

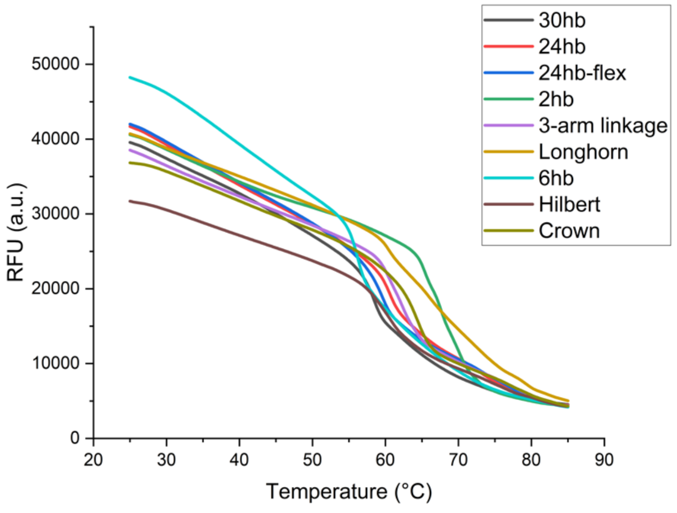

Raw data- DNA origami unfolding qPCR raw curves

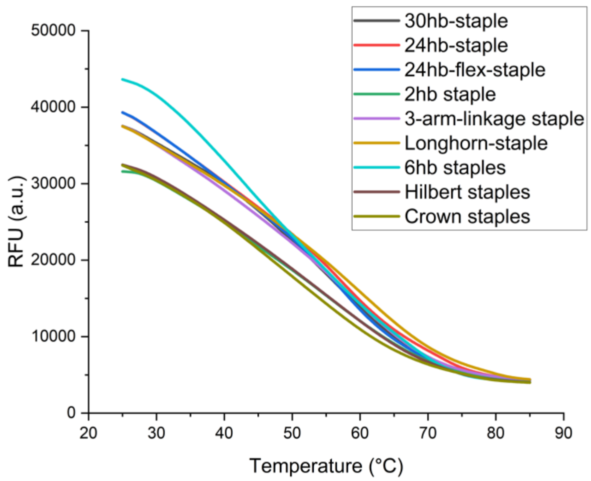

Raw data- DNA origami staple only unfolding qPCR raw curves

2. Agarose gel electrophoresis images

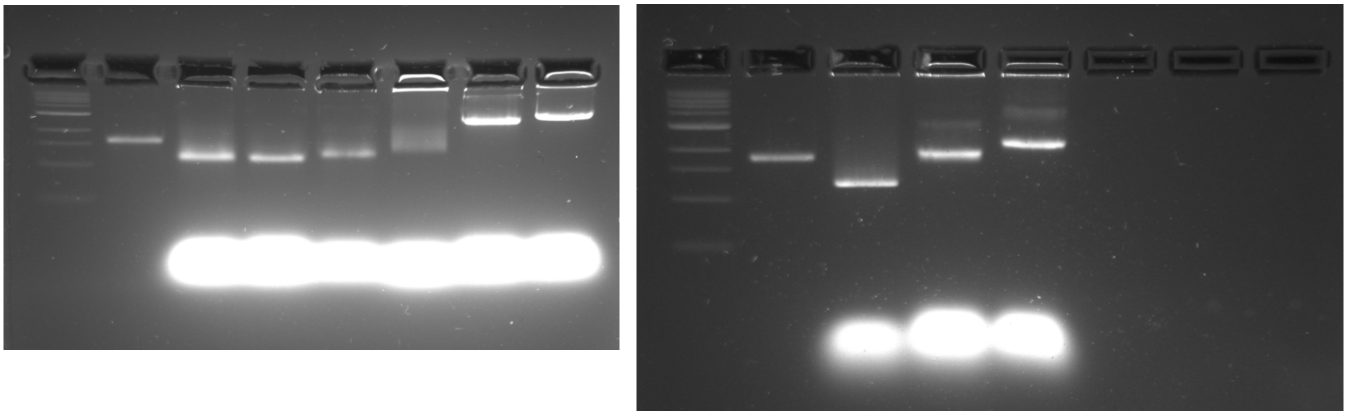

Figure S1-raw data-left image is (a) from left to right lanes there are ladder, scaffold, 30 hb, 24 hb rigid, 24 hb flex, 3 arm-linkage, Longhorn and 2 hb, and (b) is the right one from left to right in lanes there are ladder, scaffold, 6 hb, Hilber and Crown and three empty lanes. Please note that empty wells were cut in the manuscript

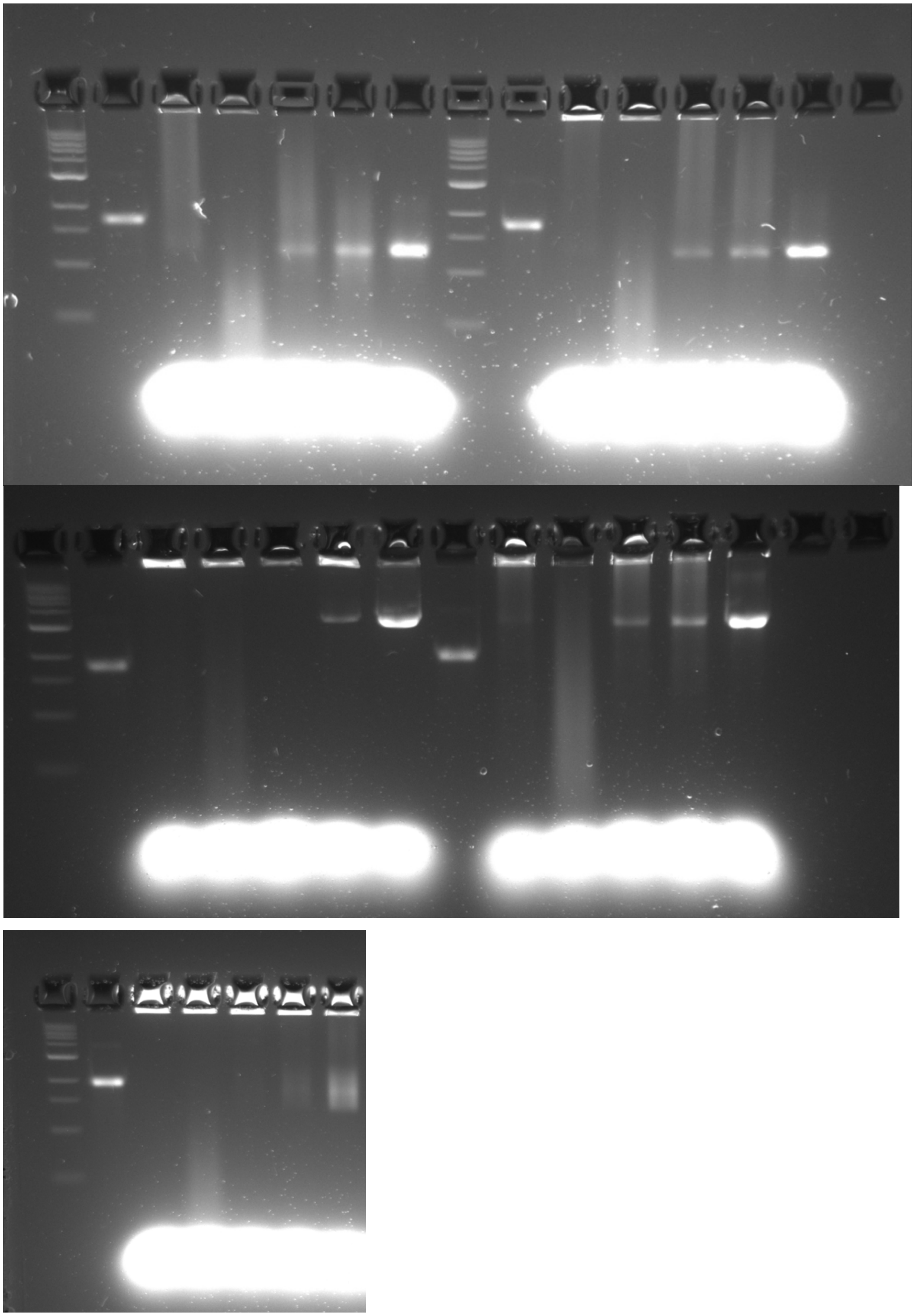

Figure S6 raw data-the top image has (a) and (b) panels (in this gel image from left to right lanes there are panel a consisted of ladder, scaffold, 1, 2, 3, 4 and 5, and then panel b starting with ladder, scaffold, 1, 2, 3, 4, 5, and empt, the middle one has (c) and (d) sections with the similar pattern and the bottom image is (e). Fol all panels from left to right lanes, there are ladder, scaffold, 1, 2, 3, 4 and 5. Please note that empty wells were cut in the manuscript.

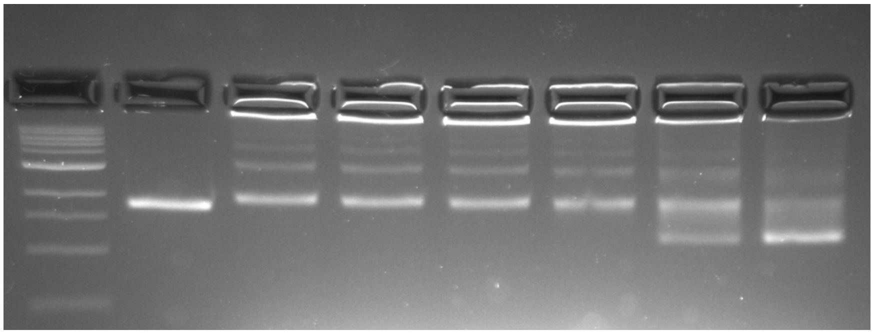

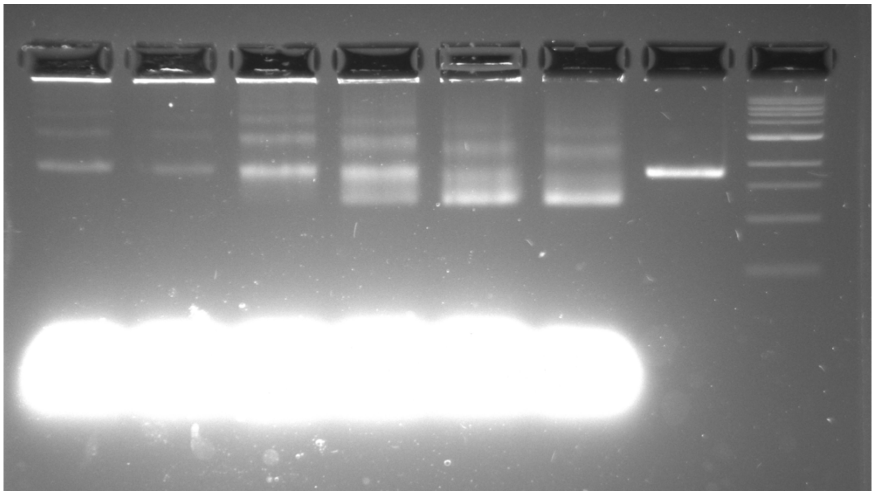

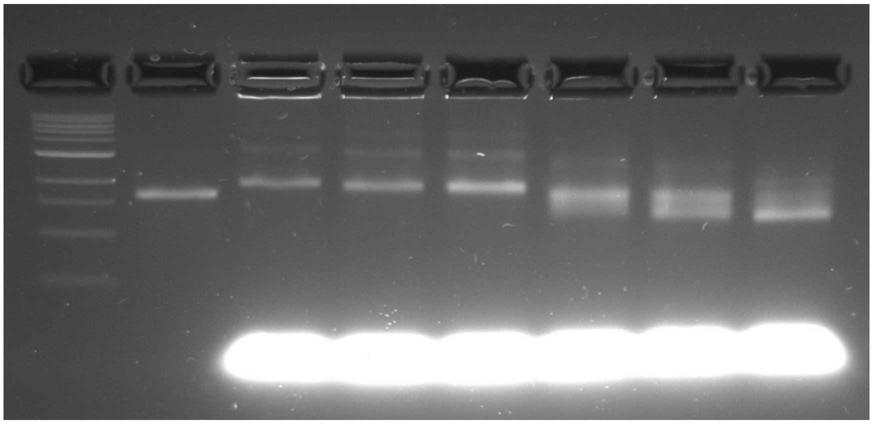

Figure S8 raw data. The top, middle and bottom images are 52, 49 and 42 ℃ gel images, respectively. The top image was captured in high magnification, so staples are not captured (however, they do not matter for the goal of this study). For the top image (52 ℃), from left to the right lanes, there are ladder, scaffold, 30s, 1, 5, 10, 30 and 60 minutes, for the middle one (49 ℃), from left to the right lanes, there are 30s, 1, 5, 10, 30, 60 minutes, scaffold and the ladder, and for the bottom image (42 ℃), there are ladder, scaffold, 0s, 30s, 1, 5, 10, and 30 minutes.

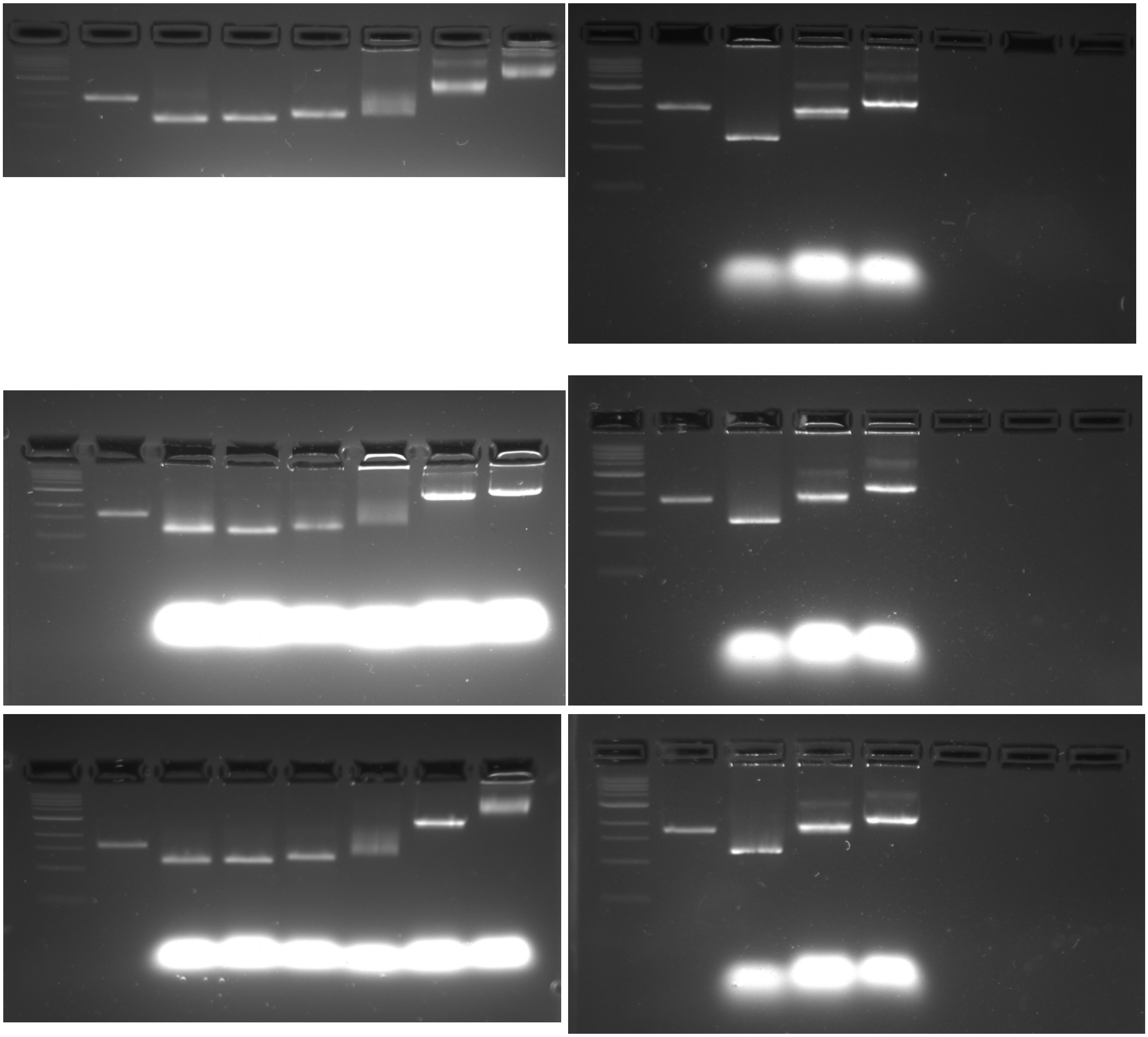

Figure S12-raw data- (a) top left and right images are the 2.5 days ramp, (b) the middle left and right images are whole qPCR images, and (c) the bottom left and right images are the focused thermal ramps. From left to right lanes in all a, b and c there are ladder, scaffold, 30 hb, 24 hb rigid, 24 hb flex, 3 arm-linkage, Longhorn and 2 hb in the left image and in the right one from left to right lanes, there are ladder, scaffold, 6 hb, Hilber and Crown. Please note that empty wells were cut in the manuscript. The top left image was captured in high magnification, so staples are not captured (however, they do not matter for the goal of this study)

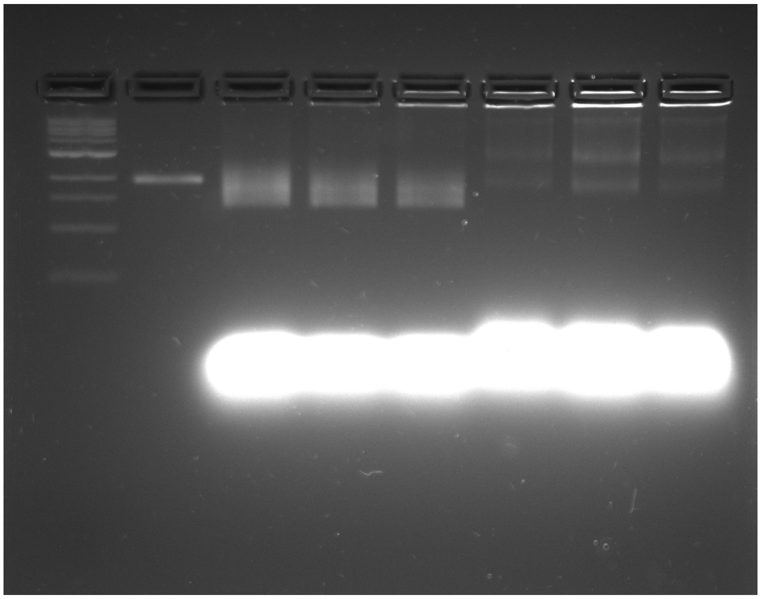

Figure 4c raw gel. From left to right lanes, there are ladder, scaffold, original design, original design, original design, longer staples, longer staples, and longer staples.

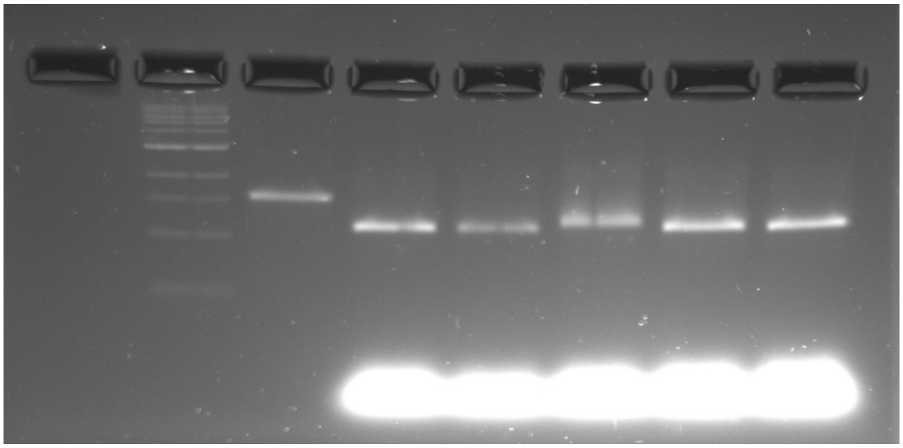

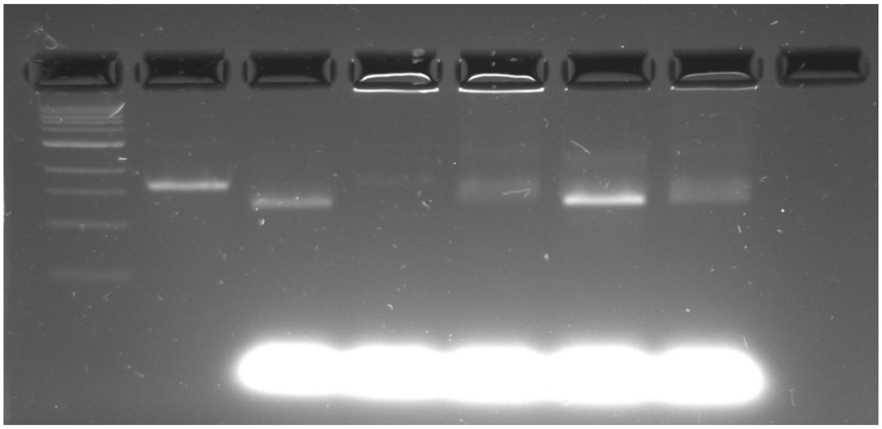

Figure 6 raw gels- (g) top image is for the 24 hb rigid structure, and lanes from left right are empty, ladder, scaffold, control, control, red dotted box, blue dotted box, and orange dotted box. (h) the bottom image is 24 hb flexible and lanes from left to right are ladder, scaffold, control, red dotted box, blue dotted box, orange dotted box and control (this lane is a repetitive control and thus was cropped in the figure, this sample was constant temperature annealing at 50 °C, which was not related to the discussion in this figure, and thus was cropped in the figure 6h). Please note that empty wells were cut in the manuscript.

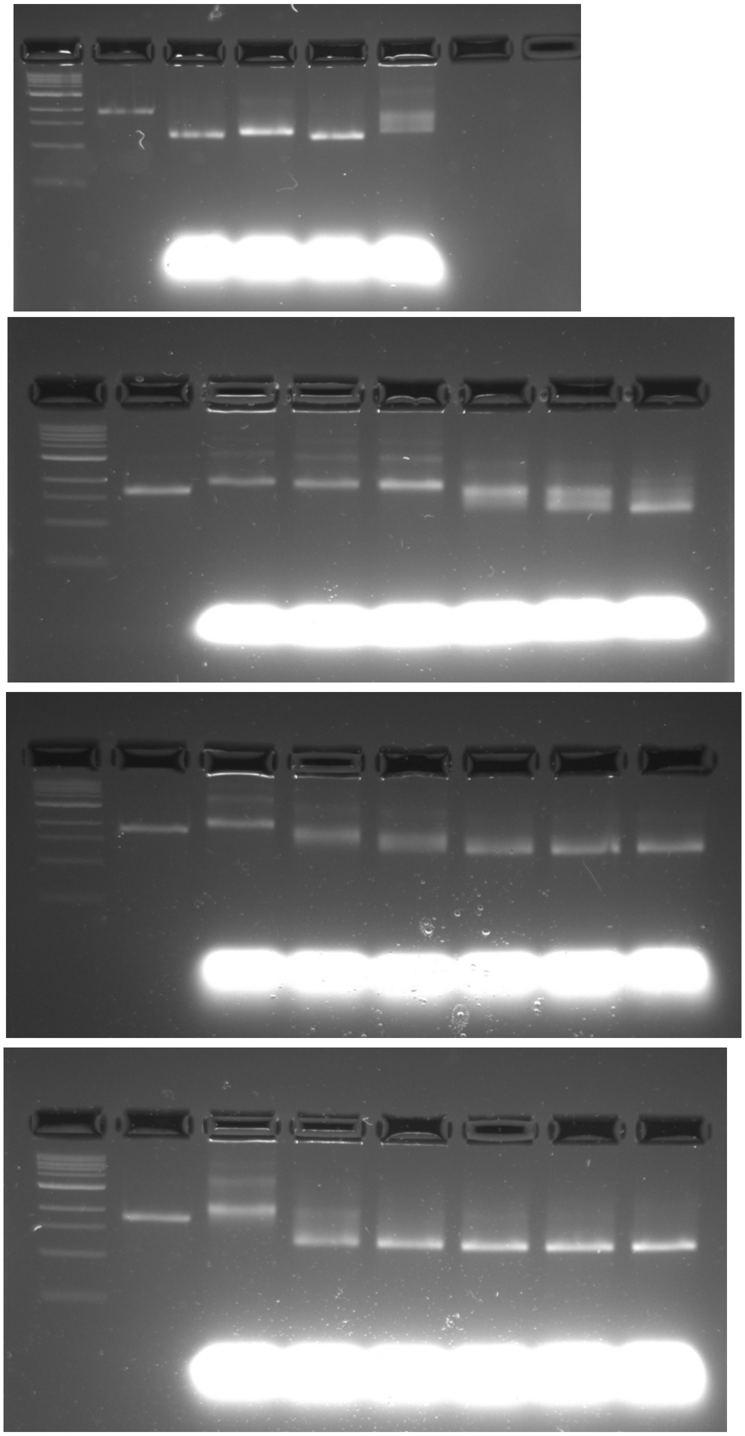

Figure 7 raw data (a) top image is panel a and from left to right lanes, there are ladder, scaffold 24 rigid 50, flex 50, rigid 55, flex 55, empty and empty. (c) the second gel from top is 30 hb, the third from the top is 24 hb-flex (d) and the bottom gel image is 24 hb rigid (e) for c, d and e from left to right lanes there are ladder, scaffold, 0, 30s, 1, 5, 10 and 30 minutes. Please note that empty wells were cut in the manuscript.

3. **Transmission Electron Microscopy images.**

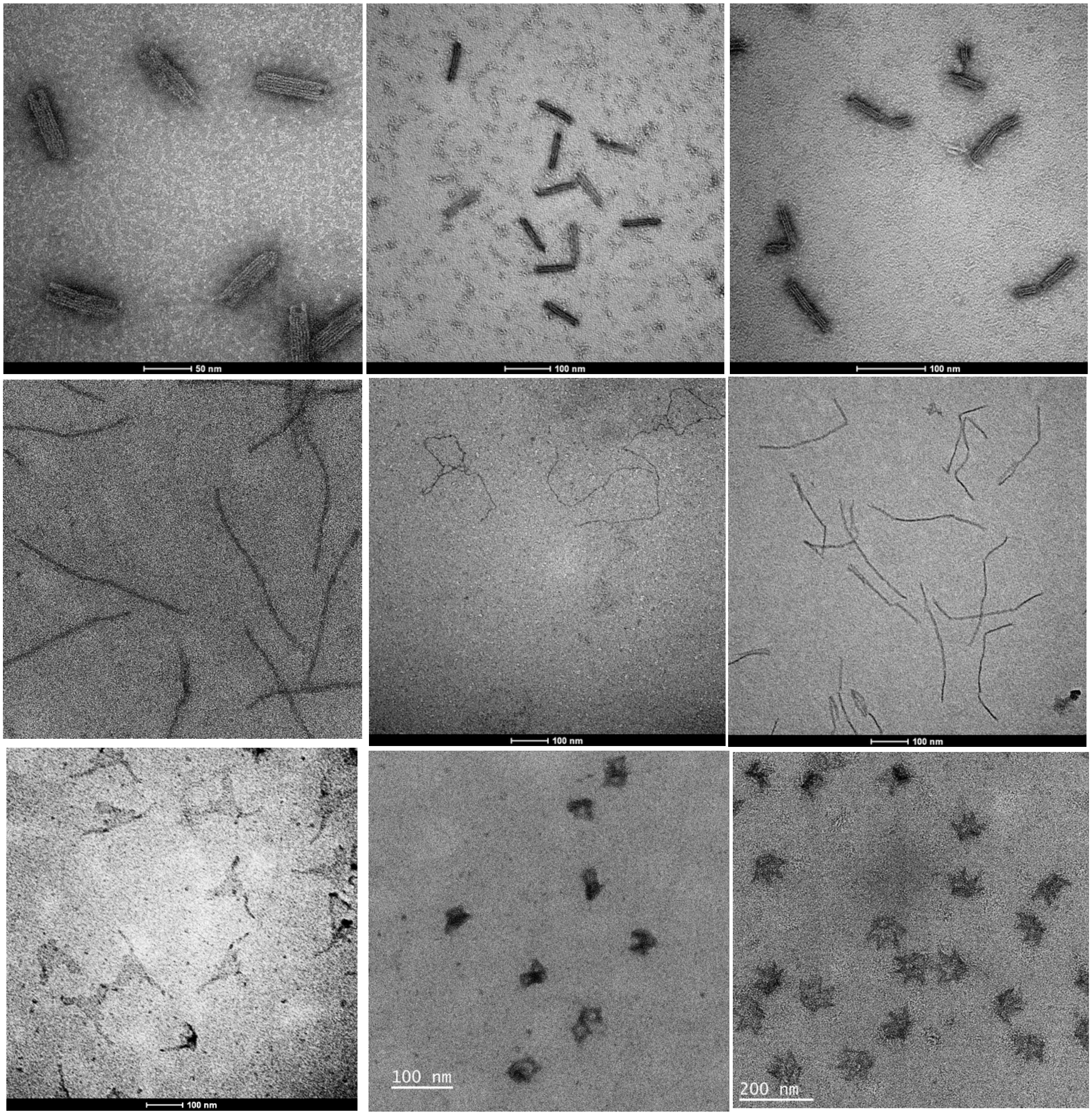

Figure 1 Raw TEM images- top row from left to right there are 30hb, 24 hb rigid, 24 hb flex, bottom row from left to right there are 6 hb, 2hb, 3 arm-linkage, and bottom row from left to right there are Longhorn logo, Hilber and Crown DNA origami.

- **Note: Figures S2, S3 and S10 are raw data and no further changes were made to them.**
